# Multi-omics reveals that NOTCH1 promotes cervical cancer progression and reduces radiosensitivity

**DOI:** 10.3389/fimmu.2025.1703032

**Published:** 2025-11-11

**Authors:** Aihua Guo, Zhixiong Su, Enhuan Zhang, Huaqin Lin, Peilin Zhong, Zhiyuan Xie, Qingzhen Zhan, Ting Ye, Yang Sun

**Affiliations:** 1 Department of Gynecology, Clinical Oncology School of Fujian Medical University, Fujian Cancer Hospital, Fuzhou, China; 2 Department of Oncology, Fujian Provincial Hospital, Fuzhou University Affiliated Provincial Hospital, Fuzhou, Fujian, China; 3 Department of Tumor Radiotherapy, Anxi County Hospital, Quanzhou, China; 4 Department of Radiation Oncology, Zhongshan Hospital, Xiamen, Fujian, China; 5 Fudan University, Xiamen, Fujian, China; 6 Clinical Research Center for Cancer Therapy, Xiamen, Fujian, China

**Keywords:** NOTCH signaling pathway, NOTCH1, has-miR-449a, cervical cancer, radiotherapy

## Abstract

**Background:**

Cervical cancer is the fourth most common malignancy in women globally. The NOTCH signaling pathway is aberrantly activated in multiple tumors, and NOTCH1, its core transmembrane receptor, is highly expressed in cervical cancer. However, NOTCH1’s mechanisms in cervical cancer progression and radiotherapy resistance, as well as its interaction with key molecules, remain unclear.

**Methods:**

This study explored the role of NOTCH1 in cervical cancer from multi-omics perspectives, including single-cell sequencing, cDNA microarrays, high-throughput sequencing, and immunohistochemistry, combined with a series of in vitro and in vivo experiments.

**Results:**

NOTCH signaling activity was negatively correlated with the overall survival and recurrence-free survival of cervical cancer patients. As the core molecule of this signaling pathway, NOTCH1 was significantly highly expressed in cervical cancer tissues and promoted cervical cancer cell proliferation in vitro. Single-cell analysis revealed that NOTCH1 was relatively highly expressed in CPA6+ and CEL+ malignant cells and involved in cell cycle regulation; further cell cycle detection assays confirmed that NOTCH1 could promote the G1-S phase transition. In addition, patients with high NOTCH1 expression showed decreased plasma cell infiltration in the microenvironment. Cell communication analysis indicated that malignant cells with high NOTCH1 expression might lead to impaired plasma cell differentiation due to the impairment of the MIF ligand-receptor pathway. Finally, NOTCH1 could reduce the radiosensitivity of cervical cancer cells to radiotherapy both in vitro and in vivo; whereas has-miR-449a, as an upstream regulatory miRNA of NOTCH1, could inhibit cervical cancer cell proliferation and enhance radiosensitivity by inhibiting NOTCH1 expression.

**Conclusions:**

This study clarifies NOTCH1’s role in promoting cervical cancer progression and reducing radiosensitivity, with has-miR-449a as a negative regulator, providing targets for optimizing cervical cancer radiotherapy.

## 1 Introduction

Cervical cancer, a life-threatening malignancy posing a severe threat to women’s health globally, has emerged as the fourth most prevalent malignant tumor among females worldwide (1). According to the World Health Organization (WHO), approximately 600,000 new cervical cancer cases were diagnosed globally in 2022, with annual deaths exceeding 300,000 (2). In recent years, the global incidence of cervical cancer has exhibited a sustained upward trend, rendering its prevention and treatment a formidable challenge (3).

The NOTCH signaling pathway is an evolutionarily highly conserved intercellular communication cascade (4, 5). Its core function lies in regulating cell fate determination, including proliferation, differentiation, apoptosis, and stem cell maintenance (6, 7). Accumulating evidence indicates that dysregulation of the NOTCH signaling pathway intricately orchestrates the initiation and progression of human hematological malignancies and solid tumors (7–13). Existing studies have demonstrated that the NOTCH signaling pathway promotes cervical cancer progression through a variety of interconnected mechanisms. It not only enhances the malignant biological behaviors of tumor cells but also participates in remodeling the tumor microenvironment (TME), creating favorable conditions for sustained tumor progression. B cells are core components of the adaptive immune system, and their developmental abnormalities or functional disorders are closely associated with autoimmune diseases, lymphomas, and other conditions (14, 15). The infiltration abundance and differentiation of B cells within the TME play crucial roles in tumor development, progression, and prognosis (16). In recent years, with the advancement of single-cell RNA sequencing (scRNA-seq) technology, the heterogeneity and functional diversity of B cells in the TME have been extensively investigated (17, 18). A growing body of evidence indicates that tumor-infiltrating B cells and plasma cells, collectively referred to as tumor-infiltrating B lymphocytes (TIL-Bs), are powerful and multifaceted participants in anti-tumor responses. They can collaborate with T cells, myeloid cells, and other immune cells to exert synergistic anti-tumor effects (19). Cynthia J Guidos et al. have shown that NOTCH signaling is involved in the development and differentiation of B cells (20). Another study revealed that NOTCH signaling promotes the ubiquitination and degradation of E2A, a transcription factor essential for B cell differentiation, thereby inhibiting B cell maturation (21). However, the regulatory roles of the NOTCH signaling pathway and its key genes in B cell infiltration and differentiation within the cervical cancer immune microenvironment remain unclear.

NOTCH1, one of the core transmembrane receptors of the NOTCH signaling pathway, is highly expressed in various tumor cells, including cervical cancer cells. By activating downstream target genes such as HES and HEY families, NOTCH1 modulates key biological processes, including tumor cell proliferation, apoptosis, angiogenesis, and TME remodeling, thereby exerting a profound impact on tumor biological behavior and treatment sensitivity (22–24). Existing research has revealed distinct roles of NOTCH1 in different malignancies: in breast cancer, NOTCH1 activation can counteract mitotic catastrophe induced by BRCA1 deficiency via restoring S/G2 and G2/M cell cycle checkpoints, thereby accelerating the malignant progression of triple-negative breast cancer (25); in colorectal cancer, inhibition of NOTCH1 expression can significantly attenuate cancer cell proliferative capacity and enhance their radiosensitivity (26). In addition, NOTCH1 has been reported to be involved in regulating the TME in various malignancies (27–29). However, the specific regulatory mechanisms of NOTCH1 in cervical cancer progression and therapeutic resistance, as well as its impact on the tumor immune microenvironment, remain to be further elucidated.

Therefore, this study employs multi-omics strategies, including scRNA-seq and high-throughput sequencing, combined with *in vitro* and *in vivo* experimental models to explore the role of NOTCH1 in cervical cancer. The objective is to further delineate the mechanism of action of NOTCH1 in cervical cancer, and provide a novel theoretical foundation and potential therapeutic targets for overcoming radiotherapy resistance and optimizing clinical treatment strategies for cervical cancer.

## 2 Materials and methods

### 2.1 Data acquisition

Twenty-five pairs of matched normal cervical and cervical cancer tissues, along with 86 cases of unmatched cervical cancer tissues, were collected from patients who underwent surgical resection at Fujian Cancer Hospital (FCH). This study also included 8 biopsy specimens of stage III cervical cancer from patients who received definitive radiotherapy for cervical cancer at Fujian Cancer Hospital (ethical approval number: K2023-142-01). In addition, this study incorporated sequencing data and clinicopathological characteristic data of 304 cervical cancer patients from The Cancer Genome Atlas (TCGA) database. The pathological information of all clinical patients is presented in **Supplementary Table 1**.

### 2.2 Single-cell data analysis

The scRNA-seq data utilized in this study was downloaded from the Gene Expression Omnibus (GEO) database (accession ID: GSE279998), which includes 16 cervical cancer tissue samples, and scRNA-seq of these samples was performed using the 10X Genomics technology (30). In scRNA-seq analysis, we filtered high-quality cells based on the following criteria: 1. The “Scrublet” package was used for doublet identification, and potential doublets were removed using a threshold of “doublet score > 0.2”. 2. “nFeature_RNA > 200 & percent.mt< 20” was applied to filter out low-quality cells. Subsequently, the “Seurat” package was used for dimensionality reduction and clustering analysis, the “harmony” package for batch effect correction, and the “FindAllMarkers” function for screening marker genes. Cell annotation was performed based on cell marker genes provided in published literature and the Cell Taxonomy website (https://ngdc.cncb.ac.cn/celltaxonomy) (31–33). The “inferCNV” package was used to distinguish between benign and malignant epithelial cells. Pseudotime analysis was conducted using the “monocle3” package, the significance of trajectory branches was validated via 100 permutation tests. Cell communication analysis was performed with the “CellChat” package, and final data visualization was achieved via the “ggplot2” package.

### 2.3 Construction of a NOTCH-related prognostic signature

Firstly, genes associated with the NOTCH signaling pathway were obtained from the MSigDB database. Subsequently, the least absolute shrinkage and selection operator (LASSO) Cox regression model was applied for dimensionality reduction and feature selection, with the optimal regularization parameter (λ) determined via 10-fold cross-validation to minimize prediction error. Based on this parameter, genes with non-zero coefficients were selected to construct the NOTCH-related prognostic signature (NRPS), and the risk score for each sample was calculated using the formula:


risk score=∑ exp(i)×coef(i)

Exp(i) denotes the expression level of gene i, and coef(i) represents the LASSO regression coefficient of gene i.

Patients were stratified into high-risk and low-risk groups according to the median risk score. Kaplan-Meier (K-M) survival analysis was performed to verify prognostic differences between the two groups, while multivariate Cox regression analysis was used to evaluate whether the signature served as an independent prognostic factor. Finally, the predictive performance was validated using Receiver operating characteristic (ROC) curves and calibration curves.

### 2.4 SHAP analysis

The SHapley Additive exPlanations (SHAP) values were used to quantify the importance of each variable in the NRPS. Firstly, the NRPS was imported into the SHAP library, and SHAP values for each gene in each sample were generated using the SHAP value calculation function; these values reflect the contribution of variables to risk stratification. The mean absolute values of SHAP values for each gene were calculated to derive a ranking of variable importance, where a higher value indicates a more significant overall impact of the gene on model predictions. Additionally, a SHAP summary plot was generated to visualize the ranking of variable importance and the trend of their influence across different expression levels, thereby intuitively demonstrating the differences in contributions of individual genes within the NRPS.

### 2.5 ssGSEA analysis

Single-sample Gene Set Enrichment Analysis (ssGSEA) is an extension of the traditional gene set enrichment analysis (GSEA) algorithm. It is primarily used for gene set enrichment scoring in individual samples, enabling direct quantification of the “activity level” of a specific gene set in a single sample. This activity level is quantified as a “score,” which facilitates comparisons of enrichment levels between different samples.

### 2.6 Cell culture

The human cervical cancer cell lines (HeLa and SiHa) and normal human cervical epithelial cell line (CP-059) were purchased from Wuhan Purcell Life Technology Co., Ltd. Their authenticity and non-contaminated status were verified by short tandem repeat (STR) authentication and mycoplasma detection. HeLa cells were cultured in Dulbecco’s Modified Eagle Medium (DMEM) supplemented with 10% heat-inactivated fetal bovine serum (FBS) and 1% penicillin-streptomycin, while CP-059 and SiHa cells were cultured in Roswell Park Memorial Institute (RPMI) 1640 medium with the same serum and antibiotic ratio. All cell lines were maintained in an incubator at 37 °C with 5% carbon dioxide (CO_2_).

### 2.7 Western blot

Cells were lysed with RIPA buffer containing protease and phosphatase inhibitor cocktails according to the manufacturer’s instructions, and protein concentration was measured and normalized by BCA assay, consistent with previous studies (34). Western Blot (WB) was performed using standard methods as described in manufacturer protocols and prior research. Antibodies used included: NOTCH1 antibody (1:1000, Rabbit monoclonal, abcam) and GAPDH antibody (1:10000, rabbit mAb (HRP), Zenbio).

### 2.8 Quantitative polymerase chain reaction

Quantitative polymerase chain reaction (qPCR) was performed as previously described (35). Total RNA was extracted from cells or tissues using TRIzol reagent (Invitrogen, USA), and its concentration was measured by NanoDrop 2000. Reverse transcription was performed on 1 μg of total RNA using the PrimeScript RT kit (TaKaRa, Japan). qPCR was carried out using SYBR Green PCR Master Mix (Applied Biosystems) on a real-time fluorescence qPCR instrument. NOTCH1 was normalized to GAPDH, while has-miR-449a was normalized to U6, with relative gene expression calculated using the 2^^-ΔΔCt^ method. Primer sequences are listed in **Supplementary Table 2**.

### 2.9 Cell transfection

Lentiviruses for NOTCH1 and has-miR-449a knockdown and overexpression, as well as their corresponding plasmids, were purchased from Han Bio Co., Ltd., Shanghai, China. The lentiviral transfection process was performed according to the manufacturer’s protocol. Cells in the logarithmic growth phase were infected with the corresponding amount of viral solution at a multiplicity of infection of 30:1, followed by incubation for 16 hours, after which the medium was replaced for further culture. Subsequently, selection was performed using puromycin, and the efficiency of the selected cells was validated by qPCR and WB.

### 2.10 Cell counting kit-8 assay

Cells in the logarithmic growth phase were trypsinized and adjusted to a concentration of 1.0×10^4^ cells/mL. Cells were seeded into 96-well plates at 100 µL per well and cultured in a 5% CO_2_, 37°C incubator for 72 hours. Before detection, 10 µL of cell counting kit-8 (CCK-8) reagent was added to each well, and after 1.5 hours of incubation, the absorbance at 450 nm optical density (OD) value was measured and recorded as the 0-hour OD value. At 24, 48, and 72 hours, 10 µL of CCK-8 reagent was added, incubated for another 1.5 hours, and OD values were measured. Cell proliferation curves were plotted based on OD values at each time point to evaluate cell proliferative activity and growth trends.

### 2.11 Colony formation assay

Logarithmically growing cells were digested and counted, then seeded into 6-well plates at a density of 500 cells/well. After cell attachment, treatment factors were added, and cells were incubated in a 37 °C, 5% CO_2_ incubator for 10–14 days, with medium changed every 3 days. When visible colonies formed, the medium was discarded, cells were washed with PBS, fixed with 4% paraformaldehyde for 15 minutes, stained with 0.1% crystal violet for 30 minutes, rinsed with water, air-dried, and photographed.

### 2.12 EdU incorporation assay

The 5-Ethynyl-2’-deoxyuridine (EdU; Elabscience) detection kit protocol was followed. Cells were seeded into 6-well plates at 1×10^5^–5×10^5^ cells/well, and after attachment. The original medium was discarded, and complete medium containing EdU was added. Cells were incubated at 37 °C for 2 hours, washed with PBS, fixed with 4% paraformaldehyde for 15 minutes, permeabilized with 0.5% Triton X-100 for 10 minutes, then incubated with click reaction solution (containing CuSO_4_, fluorescent dye Azide, etc.) in the dark for 30 minutes. After three PBS washes, cells were incubated with PBS containing 1 μg/mL Hoechst 33342 at room temperature for 10 minutes, and observed under a laser confocal microscope.

### 2.13 Wound healing assay

Logarithmically growing cells were seeded into 6-well plates. When confluence reached 90–100%, a vertical scratch was made in the central cell layer using a 200 µL sterile pipette tip. Cells were gently washed three times with PBS to remove detached cells, and serum-free medium was added. Initial scratch width was photographed at 0 hours under an inverted microscope in five fixed fields of view. After 24–48 hours of culture, photographs were taken again in the same fields, and scratch area was measured using ImageJ software.

### 2.14 Transwell invasion assay

For Transwell invasion assays, the upper surface of the chamber was pre-coated with 50 µL Matrigel (1:8 dilution, Corning) and incubated at 37 °C for 2 hours. Logarithmically growing cells were digested, resuspended in serum-free medium, and 1×10^4^ cells were seeded into the upper chamber, with 600 µL of complete medium containing 10% FBS added to the lower chamber. After 24–48 hours of incubation at 37 °C, the supernatant was discarded, and upper-layer cells were wiped off with a cotton swab. Cells were washed with PBS, fixed with 4% paraformaldehyde for 20 minutes, stained with 0.1% crystal violet for 15 minutes, photographed in five random fields of view, and the number of cells on the lower membrane was counted using ImageJ.

### 2.15 Cell cycle detection

The Cell Cycle Assay Kit (Elabscience) was used. Approximately 1×10^6^ cells were collected, washed twice with PBS, and slowly fixed with pre-cooled 70% ethanol (-20 °C) overnight at 4 °C. Before detection, ethanol was removed by centrifugation, cells were washed twice with PBS, stained with 400 µL of staining solution containing 50 µg/mL PI Reagent at room temperature in the dark for 30 minutes, and analyzed by flow cytometry.

### 2.16 Prediction of miRNA-mRNA interactions

This study used the Encyclopedia of RNA Interactomes (ENCORI) website (https://rnasysu.com/encori/, Encyclopedia of RNA Interactomes) to predict miRNAs that regulate NOTCH1. This website integrates target prediction tools such as PITA, RNA22, miRmap, DIANA-microT, miRanda, PicTar, and TargetScan. We screened potential miRNAs using the screening criterion of positive results from ≥4 prediction tools. Subsequently, we further screened the miRNAs with the highest likelihood based on the correlation of expression levels between miRNAs and NOTCH1 mRNA in TCGA cervical cancer data.

### 2.17 Dual-luciferase reporter gene assay

Using a PCR-based Accurate Synthesis (PAS)-based method, full-length overlap primers were designed, and NOTCH1 (NM_017617.5)-WT (wild type) and NOTCH1 (NM_017617.5)-MUT (mutant) sequences were synthesized via two rounds of PCR, then cloned into the psicheck2.0 vector to obtain NOTCH1-WT and NOTCH1-MUT recombinant vectors. These vectors were co-transfected with miRNA mimic or control NC into HeLa/SiHa cells. After 48 hours of transfection, the medium was discarded, cells were washed with PBS, lysed on ice with lysis buffer for 15 minutes, and 20 µL of cell lysate supernatant was added to a 96-well microplate. 100 µL of luciferase detection reagent was added to measure firefly luciferase activity, followed by 100 µL of Stop&Glo reagent to measure renilla luciferase activity. Luminescence values were read by a microplate reader, with relative fluorescence intensity calculated by normalizing firefly luciferase activity to renilla luciferase activity.

### 2.18 Immunohistochemistry

Tissue sections were routinely dewaxed to water, and antigen retrieval was performed by high-temperature and high-pressure treatment with ethylenediaminetetraacetic acid (EDTA) repair buffer (pH 9.0). Endogenous peroxidase activity was blocked by incubating with 3% hydrogen peroxide solution at room temperature for 15 minutes, followed by three PBS washes. Sections were blocked with 5% BSA at room temperature for 1 hour, then incubated with Ki67 (1:200 dilution) and NOTCH1 (1:150 dilution) primary antibodies overnight at 4 °C. The next day, sections were warmed to room temperature for 30 minutes, washed three times with PBS, incubated with biotin-labeled secondary antibody at room temperature for 1 hour, washed again with PBS, and incubated with streptavidin-horseradish peroxidase complex at room temperature for 30 minutes. Color development was performed with 3,3’-Diaminobenzidine chromogenic solution (DAB) chromogenic solution (monitored microscopically to control development intensity), followed by hematoxylin counterstaining, gradient ethanol dehydration, xylene clearing, and neutral gum mounting. Sections were scanned at full field of view and saved as multi-magnification digital images.

### 2.19 Animal experiments

Logarithmically growing HeLa cells transfected with different lentiviral treatments (1×10^7^ cells/100 µL) were resuspended in PBS containing 50% Matrigel (Corning) and subcut aneously injected into the right dorsum of 4-week-old female Balb/c -nude mice (100 µL per mouse). Mice were randomly divided into four groups: control (has-miR-449a-Vector + NOTCH1-Vector group), has-miR-449a-vector + NOTCH1-Vector + radiotherapy group, has-miR-449a-oe + NOTCH1-vector + radiotherapy group, and has-miR-449a-oe + NOTCH1-oe + radiotherapy group, with 5 mice per group. When tumor volume reached 80–100 mm³, local irradiation was administered using a small animal radiotherapy machine (total dose 10 Gy, divided into 5 fractions of 2 Gy daily). Tumor length (L) and width (W) were measured with vernier calipers every 7 days, and tumor volume was calculated using the formula V=0.5×L×W² to plot growth curves. Upon reaching a tumor volume of 1000 mm³ or exhibiting a moribund status, mice were euthanized. Immediately after confirmation of death, tumors were surgically excised and weighed. The animal experiments in this study were approved by the Institutional Animal Care and Use Committee (IACUC) of Fujian Medical University (Ethics Number: IACUC FIMU 2024-Y-1928).

### 2.20 Statistical analysis

Data are presented as Mean ± SD for quantitative variables, analyzed using GraphPad Prism 9.0 and Rstudio 4.1.0. Independent samples t-test was used for comparisons between two groups; one-way ANOVA for multiple groups; and Spearman rank correlation test for correlation analysis. Repeated-measure tumor volume data in *in vivo* experiments were analyzed by two-way ANOVA, with time and treatment factors as variables to assess intergroup differences and interactions. All tests were considered statistically significant at P<0.05, and experimental data were validated in three independent replicates.

## 3 Result

### 3.1 Clinical significance of the NOTCH signaling pathway in cervical cancer

**Figure 1:**
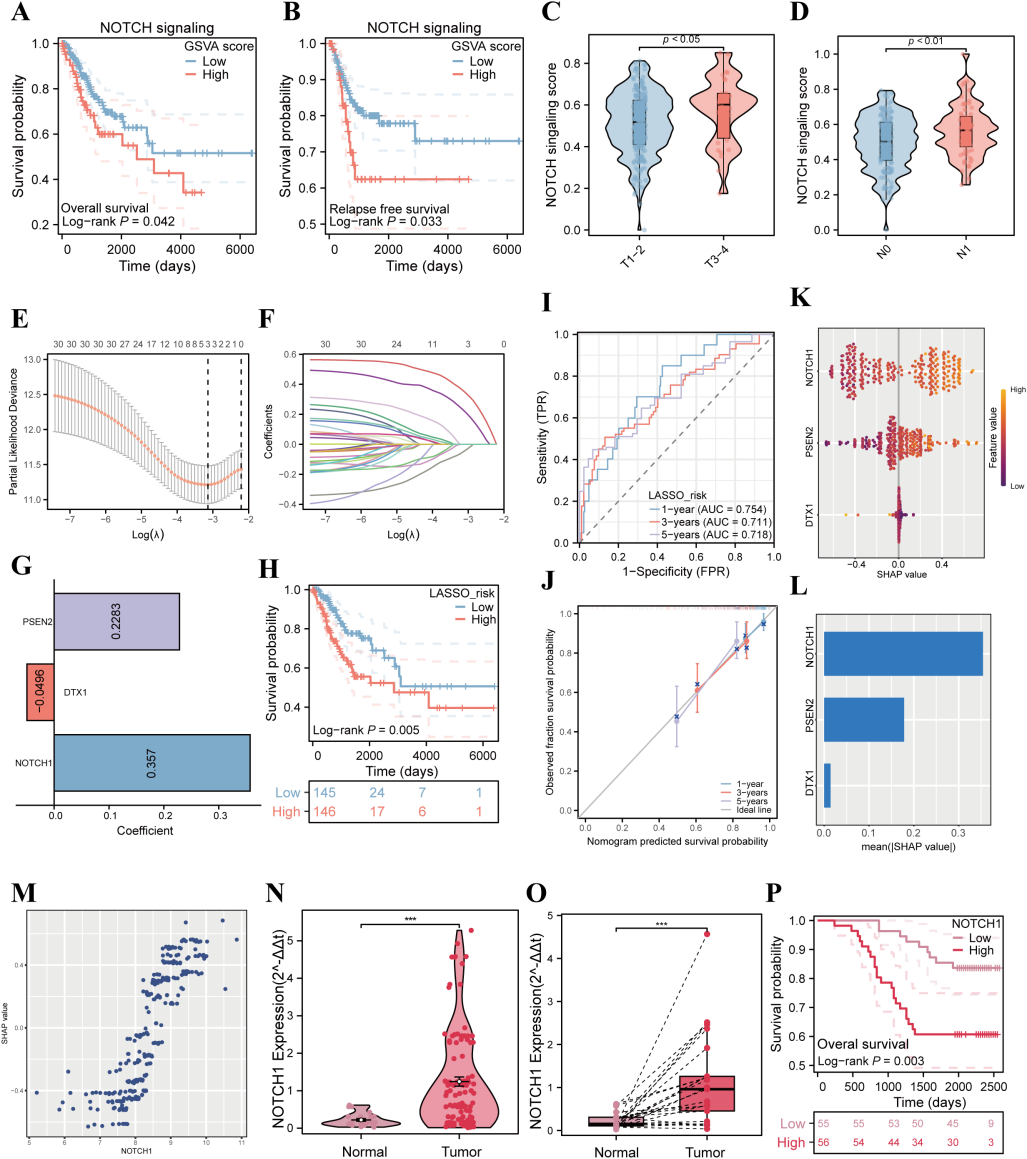
Correlation analysis of NOTCH signaling pathway with poor prognosis in cervical cancer patients. **(A)** K-M curves analyze the correlation between the ssGSEA score of the NOTCH signaling pathway and OS of patients in the TCGA cervical cancer cohort. **(B)** K-M curves analyze the correlation between the ssGSEA score of the NOTCH signaling pathway and RFS of patients in the TCGA cervical cancer cohort. **(C)** Violin plot compares the differences in the ssGSEA score of the NOTCH signaling pathway among cervical cancer patients with different T stages. **(D)** Violin plot compares the differences in the ssGSEA score of the NOTCH signaling pathway among cervical cancer patients with different N stages. **(E)** LASSO coefficient profiles of the NOTCH signaling pathway-related genes. **(F)** Plots of the tenfold cross-validation error rates. **(G)** The coefficients of three candidate genes. **(H)** Kaplan–Meier survival curve of OS between the high- and low-risk groups in TCGA cohort. **(I)** The ROC curve of the NRPS in TCGA cohort. **(J)** Calibration curves showing the concordance between predicted and observed 1-, 3-, and 5-year survival rates. **(K)** SHAP analysis summary plot showing the importance ranking of the three variables. **(L)** The importance ranking of the three variables according to the mean (|SHAP value|). **(M)** The scatter plot illustrates the relationship between NOTCH1 mRNA expression and SHAP values. **(N)** Violin plot compares the differences in NOTCH1 mRNA expression between normal cervical tissues and cervical cancer tissues in unpaired clinical samples. **(O)** Violin plot compares the differences in NOTCH1 mRNA expression between normal cervical tissues and cervical cancer tissues in paired clinical samples. **(P)** K-M curves analyze the correlation between NOTCH1 expression level and OS of cervical cancer patients in the Fujian Cancer Hospital (FCH) cohort. ***: p<0.001.

To quantify the impact of the activation level of the NOTCH signaling pathway on the prognosis of cervical cancer, we calculated the NOTCH signaling pathway score for each sample in the TCGA database using ssGSEA. Further K-M analysis results showed that the NOTCH signaling pathway score was significantly associated with poorer overall survival (OS) and recurrence-free survival (RFS) in cervical cancer patients (all p<0.05; **Figures 1A, B**). In addition, results of subgroup difference analysis indicated that the activation level of the NOTCH signaling pathway in patients with stage T3–4 was significantly higher than that in patients with stage T1-2, and the activation level in patients with stage N1 was also significantly higher than that in patients with stage N0 (all p<0.05; **Figures 1C, D**).

To further investigate the impact of NOTCH signaling pathway-related genes on the prognosis of cervical cancer, we employed LASSO analysis for variable selection and constructed the NRPS. After screening, NOTCH1, DTX1, and PSEN2 were identified as candidate genes, collectively forming the NRPS with model coefficients of 0.357, -0.0496, and 0.2283, respectively (**Figures 1E-G**). Subsequently, patients from the TCGA database were stratified into high-risk and low-risk groups using the median risk score as the cutoff value. K-M curve analysis revealed that the prognosis of the high-risk group was significantly poorer than that of the low-risk group (p<0.05, **Figure 1H**). ROC curve analysis demonstrated that the NRPS exhibited good predictive performance for 1-year, 3-year, and 5-year overall survival rates of cervical cancer patients, with corresponding area under the curve (AUC) values of 0.754, 0.711, and 0.718, respectively (**Figure 1I**); results from the calibration curve also confirmed the predictive accuracy of this signature (**Figure 1J**). Moreover, The results of univariate and multivariate analyses demonstrated that NRPS is an independent risk factor for OS in patients with cervical cancer (p<0.05; HR = 4.644, 95%CI: 2.013 - 10.712; **Supplementary Table 3**). Additionally, to clarify the contribution of each variable in constructing the NRPS to its outcomes, we performed SHAP analysis, which showed that NOTCH1 had the highest absolute SHAP value (**Figures 1K-M**), suggesting that NOTCH1 may play a central role in the process by which the NOTCH pathway influences the prognosis of cervical cancer patients.

In addition, qPCR experiments were performed using cervical cancer cDNA microarrays. The results showed that in both paired and unpaired samples, the expression of NOTCH1 in tumor tissues was significantly higher than that in normal cervical tissues (all p<0.05; **Figures 1N, O**). Finally, patients were divided into high and low expression groups based on the median value of NOTCH1 expression, and K-M survival analysis was performed. The results indicated that the OS of the high NOTCH1 expression group was significantly worse than that of the low expression group in FCH cohort (p<0.05; **Figure 1P**).

### 3.2 NOTCH1 promotes cervical cancer cell proliferation *in vitro*

**Figure 2:**
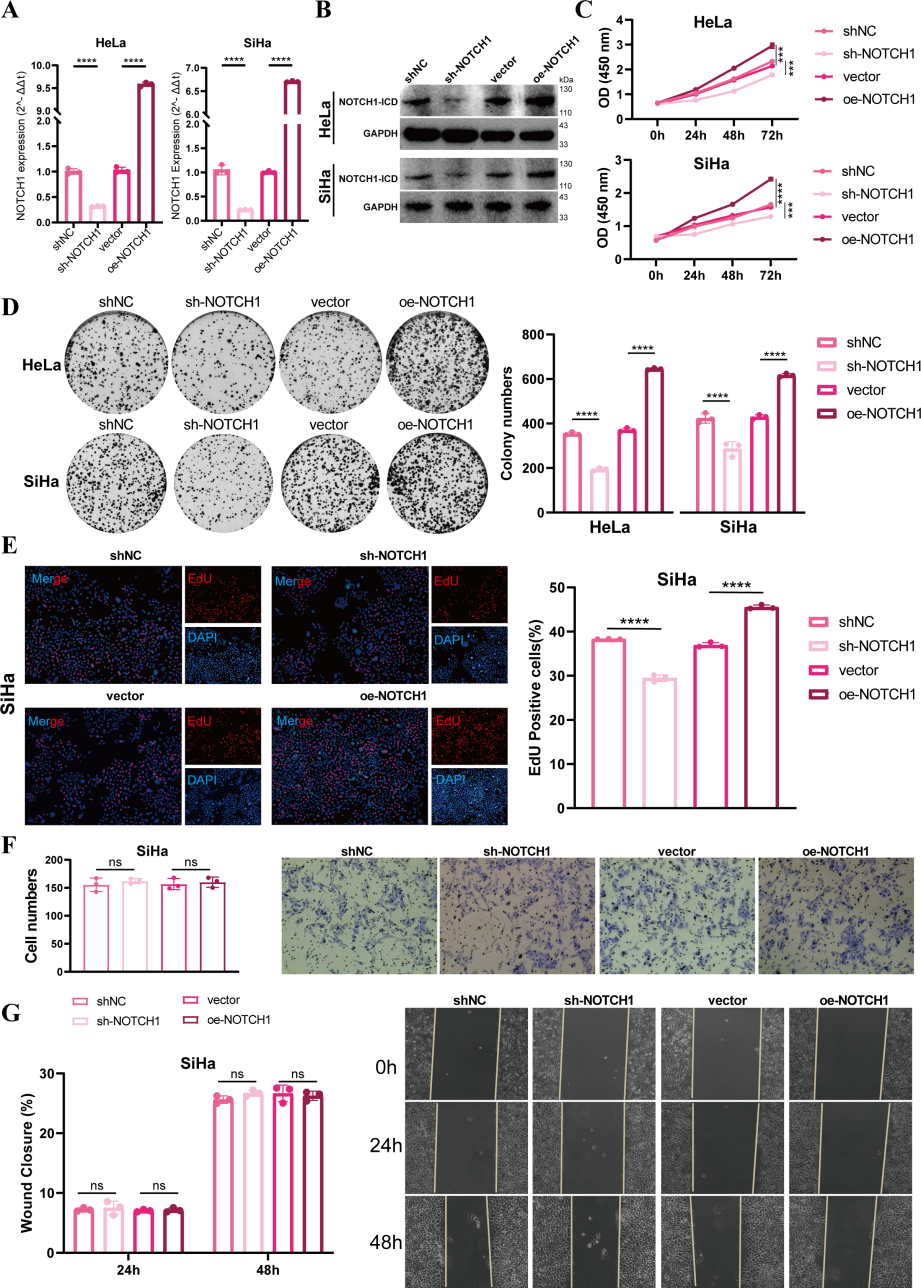
NOTCH1 promotes cervical cancer cell proliferation *in vitro*. **(A)** Bar graphs show the qPCR results of NOTCH1 mRNA in HeLa and SiHa cell lines after different interventions (n=3). **(B)** WB images show NOTCH1-ICD protein levels in HeLa and SiHa cell lines after different interventions (n=3). **(C)** Line graphs show the CCK-8 assay results of HeLa and SiHa cell lines after different interventions (n=3). **(D)** Colony formation assay results and representative images of HeLa and SiHa cell lines after different interventions (n=3). **(E)** EdU incorporation assay results and representative images of SiHa cell lines after different interventions (n=3). **(F)** Transwell assay results and representative images of SiHa cell lines after different interventions (n=3). **(G)** Wound healing assay results and representative images of SiHa cell lines after different interventions (n=3). NOTCH1-ICD: NOTCH1 Intracellular Domain. ns: p>0.05; ***: p<0.001; ****: p<0.0001.

Results of qPCR and WB demonstrated that we successfully established NOTCH1 knockdown and overexpression HeLa and SiHa cell lines via lentiviral transfection (all p<0.05; **Figures 2A, B**). To evaluate cell proliferation ability, we performed CCK-8, colony formation, and EdU incorporation assays. The results consistently showed that the proliferation ability of HeLa and SiHa cells was significantly inhibited after NOTCH1 knockdown, whereas the opposite effect was observed in the NOTCH1 overexpression groups (all p<0.05; **Figures 2C–E**; **Supplementary Figure 1A**). However, results of transwell assay and wound healing assay indicated that no significant changes in the migration and invasion abilities of HeLa and SiHa cells were observed after NOTCH1 knockdown or overexpression (all p>0.05; **Figure 2F, G**; **Supplementary Figure 1B, C**).

### 3.3 NOTCH1 regulates the cell cycle

**Figure 3:**
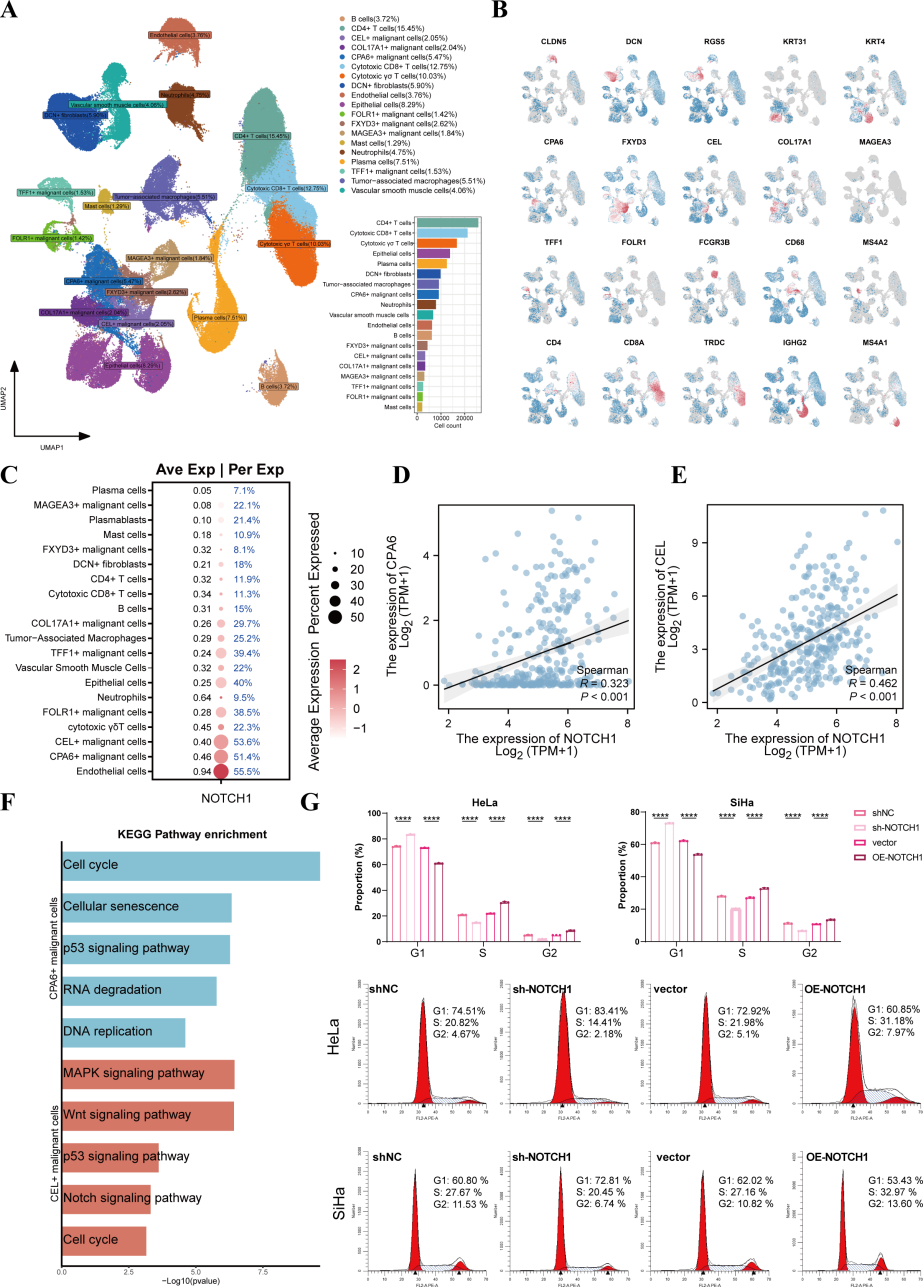
NOTCH1 is involved in regulating the cell cycle of cervical cancer cells. **(A)** UMAP plot shows the distribution of different cell subsets. **(B)** UMAP plot shows the distribution of marker genes in different cell subsets. **(C)** Bubble plot shows the expression of NOTCH1 in different cell subsets. **(D, E)** Scatter plots show the expression correlation between NOTCH1 and CPA6, as well as between NOTCH1 and CEL, in the TCGA cervical cancer cohort. **(F)** Bar graph shows the results of KEGG enrichment analysis of CPA6+ malignant cells and CEL+ malignant cells. **(G)** Cell cycle detection assay results of HeLa and SiHa cells (n=3). UMAP: Uniform Manifold Approximation and Projection. ****: p<0.0001.

To further explore the expression of NOTCH1 at the single-cell level, we performed scRNA-seq analysis. Through dimensionality reduction, clustering, and annotation, more than 160,000 cells obtained from 16 cervical cancer samples were divided into 19 subpopulations (**Figure 3A**), and **Figure 3B** presents the marker genes of each subpopulation. Among these, epithelial cells were classified into COL17A1+ malignant cells, CPA6+ malignant cells, FLOR1+ malignant cells, FXYD3+ malignant cells, MAGEA3+ malignant cells, TFF1+ malignant cells, and benign epithelial cells based on inferCNV results and specifically highly expressed genes (**Supplementary Figure 2**).

The bubble plot showed that NOTCH1 was expressed in most cells, with the highest expression observed in endothelial cells, CPA6+ malignant cells, and CEL+ malignant cells (**Figure 3C**). Results of expression correlation analysis in the TCGA cohort indicated that the mRNA expression of NOTCH1 was significantly positive correlated with that of CPA6 and CEL (all p<0.05; **Figures 3D, E**). Therefore, we conducted enrichment analysis on CPA6+ malignant cells and CEL+ malignant cells, and the results showed that both of these two malignant cell subpopulations were significantly enriched in the cell cycle and classical oncogenic pathways (all p<0.05; **Figure 3F**).

To verify the effect of NOTCH1 on the cell cycle, we performed a cell cycle detection assay. The results demonstrated that after NOTCH1 knockdown, the proportion of cells in the G1 phase significantly increased, while the proportions of cells in the S phase and G2 phase significantly decreased; whereas the opposite trend was observed in the NOTCH1 overexpression groups (all p<0.05; **Figure 3G**). This indicates that NOTCH1 can promote the G1-to-S phase transition of cervical cancer cells, thereby promoting the proliferation of cervical cancer cells.

### 3.4 NOTCH1 affects plasma cell maturation

**Figure 4:**
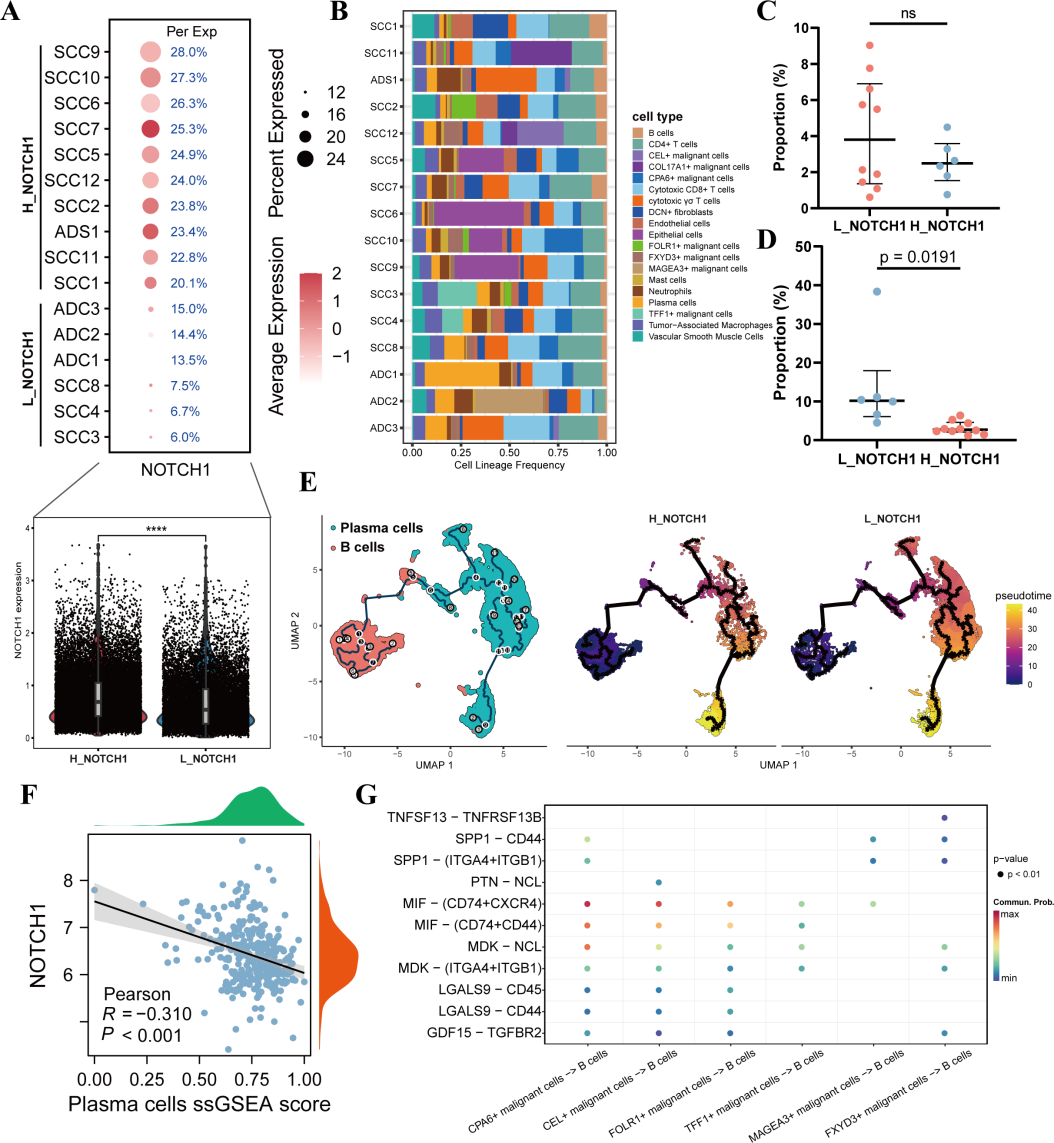
NOTCH1 affects the differentiation of B cells into plasma cells. **(A)** Differences in NOTCH1 expression between different samples, as well as between the H_NOTCH1 group and L_NOTCH1 group. **(B)** Plot of immune infiltration proportions among different single-cell samples. **(C)** Difference in the proportion of B cells between the H_NOTCH1 group and L_NOTCH1 group. **(D)** Difference in the proportion of plasma cells between the H_NOTCH1 group and L_NOTCH1 group. **(E)** Results of pseudotime analysis of B cells and plasma cells. **(F)** Scatter plot shows the correlation between NOTCH1 and plasma cells ssGSEA score. **(G)** Bubble plot shows the ligand-receptor interactions between different malignant cells and B cells. ns: p>0.05.

To further explore the impact of NOTCH1 on the cervical cancer immune microenvironment, 16 single-cell samples were first divided into the L_NOTCH1 group (low NOTCH1 expression) and H_NOTCH1 group (high NOTCH1 expression) based on the expression level of NOTCH1 in each sample. The violin plot confirmed that the expression of NOTCH1 in the H_NOTCH1 group was significantly higher than that in the L_NOTCH1 group (p<0.05; **Figure 4A**). Subsequently, we analyzed the proportion of various cell subpopulations in each sample. The results showed that compared with the L_NOTCH1 group, the H_NOTCH1 group had no significant difference in the infiltration proportion of B cells (p>0.05; **Figures 4B, C**), while the infiltration proportion of plasma cells was significantly reduced (p<0.05; **Figures 4B, D**). Further pseudotime analysis results revealed that the ability of B cells to differentiate into plasma cells was decreased in the H_NOTCH1 group (**Figure 4E**). By screening the marker genes of plasma cells for ssGSEA, the results indicated that in the TCGA cervical cancer cohort, the plasma cell ssGSEA score was significantly negatively correlated with NOTCH1 expression (R=-0.310; p<0.05; **Figure 4F**). Finally, results of cell-cell communication analysis showed that there were significant differences in the interaction of MIF-(CD74+CXCR4) and MIF-(CD74+CD44) secretory ligand-receptor pathways between malignant cells with high NOTCH1 expression and those with low NOTCH1 expression (**Figure 4G**). This suggests that tumor cells with high NOTCH1 expression may have restricted B cell differentiation due to the inhibition of these ligand-receptor pathways.

### 3.5 has-miR-449a inhibits NOTCH1 expression

**Figure 5:**
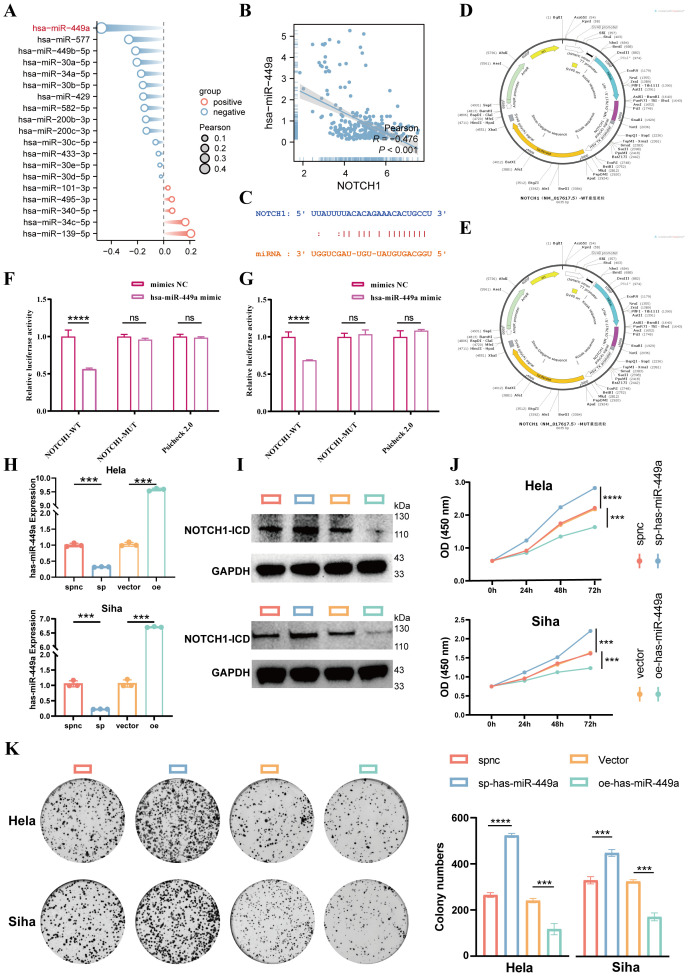
Has-miR-449a targets and inhibits NOTCH1 expression to suppress cervical cancer cell proliferation. **(A)** Lollipop plot shows the expression correlation between 19 candidate miRNAs and NOTCH1. **(B)** Scatter plot shows the expression correlation between NOTCH1 and has-miR-449a in the TCGA cervical cancer cohort. **(C)** Predicted binding genomic sequences between NOTCH1 and has-miR-449a. **(D)** Schematic diagram of NOTCH1-WT plasmid construction. **(E)** Schematic diagram of NOTCH1-MUT plasmid construction. **(F)** Bar graph shows the results of dual-luciferase reporter gene assay in HeLa cell line (n=3). **(G)** Bar graph shows the results of dual-luciferase reporter gene assay in SiHa cell line (n=3). **(H)** Bar graph shows qPCR verifying the lentiviral transfection efficiency of has-miR-449a (n=3). **(I)** WB shows NOTCH1-ICD expression after different has-miR-449a interventions (n=3). **(J)** Line graph shows the CCK-8 assay results of HeLa and SiHa cells after different interventions (n=3). **(K)** Colony formation assay results and representative images of HeLa and SiHa cells after different interventions (n=3). NOTCH1-ICD: NOTCH1 Intracellular Domain. ***: p<0.001; ****: p<0.0001.

MicroRNAs (miRNAs) are one of the key factors contributing to the dysregulation of gene expression in cancer; therefore, we further explored the miRNAs that regulate NOTCH1 expression. First, via screening on the ENCORI website (a comprehensive platform for ceRNA interaction analysis), a total of 19 miRNAs potentially involved in regulating NOTCH1 were identified. Results of correlation analysis showed that has-miR-449a exhibited the strongest negative correlation with NOTCH1 mRNA expression (R=-0.476; p<0.01; **Figures 5A, B**). Subsequently, we designed NOTCH1-WT (wild-type NOTCH1) and NOTCH1-MUT (mutant NOTCH1) plasmids based on their interaction sites (**Figures 5C–E**). Results of the dual-luciferase reporter assay demonstrated that has-miR-449a could target NOTCH1 and thereby inhibit its expression (all p<0.05; **Figures 5F, G**). Then, the expression of hsa-miR-449a was significantly decreased in cervical cancer cell lines (HeLa and SiHa) compared with that in normal cervical epithelial cells (CP-H059) (p<0.05; **Supplementary Figure 3**).

In addition, we used a has-miR-449a sponge lentivirus (to sequester and inhibit has-miR-449a activity) and a has-miR-449a precursor overexpression lentivirus (to upregulate has-miR-449a expression) for cell transfection. qPCR was performed to verify the transfection efficiency, and the results confirmed successful modulation of has-miR-449a expression (all p<0.05; **Figure 5H**). Results of WB showed that inhibition of has-miR-449a expression led to an increase in NOTCH1 protein expression, whereas overexpression of has-miR-449a resulted in a decrease in NOTCH1 protein expression (**Figure 5I**). These results indicate that NOTCH1 is a regulatory target gene of has-miR-449a.

Finally, results of CCK-8 and colony formation assays demonstrated that inhibition of has-miR-449a expression significantly enhanced the proliferation ability of cervical cancer cells, while the opposite effect was observed with has-miR-449a overexpression. This indicates that has-miR-449a can inhibit the proliferation of cervical cancer cells via inhibited NOTCH1 expression *in vitro* (all p<0.05; **Figures 5J, K**).

### 3.6 NOTCH1 affects radiosensitivity

**Figure 6:**
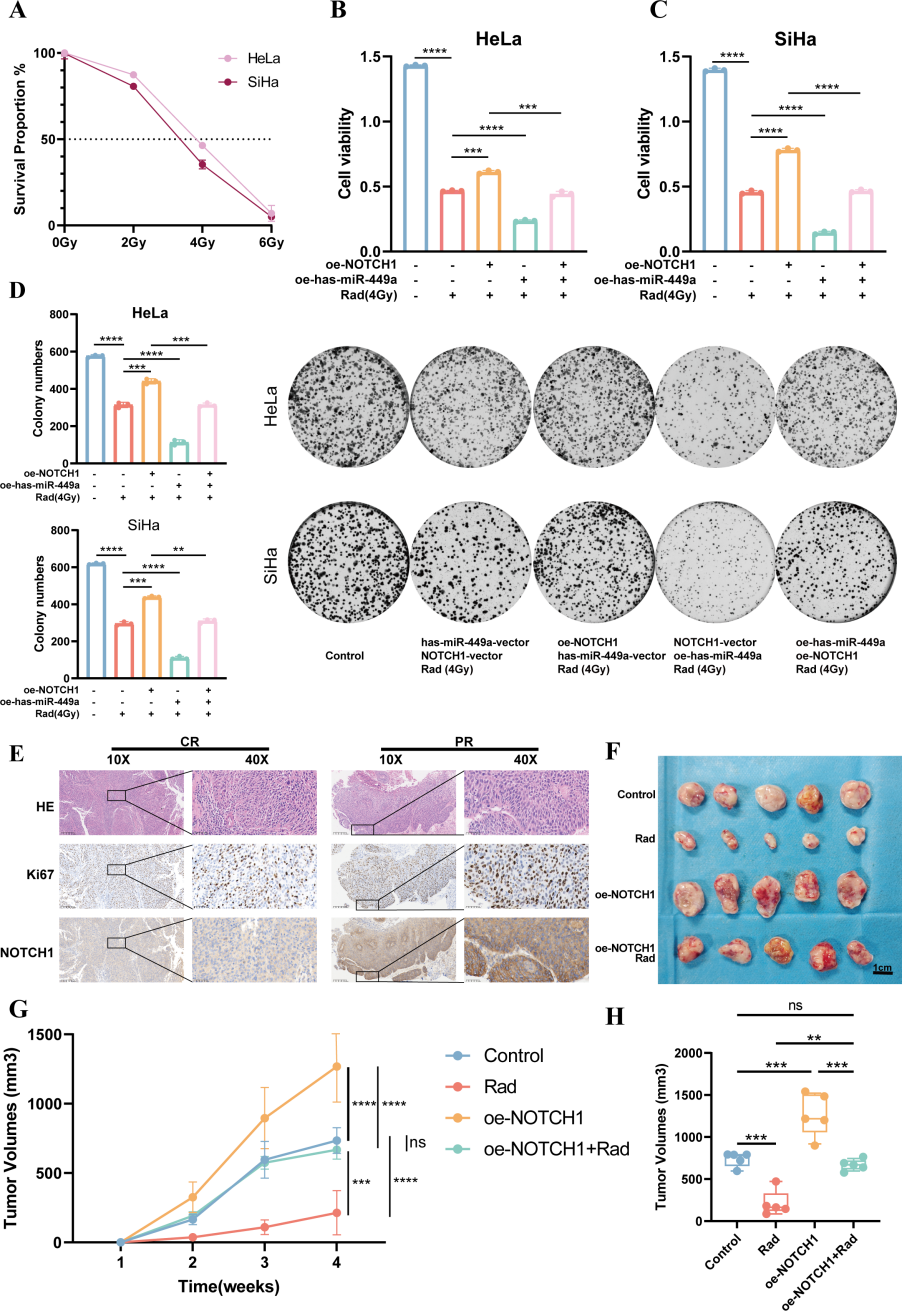
NOTCH1 reduces the radiosensitivity of cervical cancer cells. **(A)** Line graph shows the survival rates of HeLa and SiHa cells after exposure to different radiation doses (n=3). **(B)** Bar graph shows the CCK-8 assay results of HeLa cells under different conditions (n=3). **(C)** Bar graph shows the CCK-8 assay results of SiHa cells under different conditions (n=3). **(D)** Colony formation assay results and representative images of HeLa and SiHa cells under different conditions (n=3). **(E)** Representative immunohistochemistry images of cervical cancer patients with different treatment responses after definitive radiotherapy. **(F)** Results of subcutaneous tumor-bearing mouse assay (n=5). **(G)** Line graph shows the growth curves of subcutaneous tumors in mice under different conditions (n=5). **(H)** Box plot shows the volume differences of subcutaneous tumors in mice under different conditions (n=5). ns: p>0.05; **: p<0.01; ***: p<0.001; ****: p<0.0001.

Radiotherapy is one of the key treatment modalities for cervical cancer. Previous studies have shown that both has-miR-449a and the NOTCH signaling pathway are associated with radiosensitivity. Therefore, in this study, we further explored the relationship between NOTCH1 and the radiosensitivity of cervical cancer. First, results of the CCK-8 assay showed that 4 Gy was the half-maximal inhibitory concentration (IC50) for *in vitro* irradiation of HeLa and SiHa cell lines (**Figure 6A**). Thus, 4 Gy was used as the radiotherapy intervention in subsequent *in vitro* experiments.

Results of the cytotoxicity assay demonstrated that compared with the group receiving 4 Gy irradiation alone (Rad group), the cell viability was significantly increased in the NOTCH1 overexpression plus radiotherapy group (oe-NOTCH1+Rad group) and significantly decreased in the has-miR-449a overexpression plus radiotherapy group (oe-has-miR-449a+Rad group). Furthermore, compared with the oe-NOTCH1+Rad group, the cell viability was significantly decreased in the group with concurrent overexpression of NOTCH1 and has-miR-449a plus radiotherapy (oe-NOTCH1+oe-has-miR-449a+Rad group). These results indicate that NOTCH1 can reduce the *in vitro* radiosensitivity of cervical cancer cells, while has-miR-449a can increase the *in vitro* radiosensitivity of cervical cancer cells by inhibiting NOTCH1 expression (all p<0.05; **Figures 6B–D**). Consistently, the colony formation assay yielded the same conclusion.

In addition, we collected cervical cancer tissues from patients with cervical cancer who received definitive radiotherapy. Results of IHC staining showed that patients with partial response (PR) had higher protein expression levels of Ki67 and NOTCH1 than complete response (CR) patients (**Figure 6E**). Finally, we established a subcutaneous tumor-bearing mouse model to verify the effect of NOTCH1 on radiosensitivity *in vivo*. The results showed that compared with the control group, the tumor volume and growth rate in the radiotherapy-only group (Rad) were significantly reduced, while those in the NOTCH1 overexpression group (oe-NOTCH1) were significantly increased. Moreover, compared with the Rad group, the tumor volume and growth rate in the oe-NOTCH1+Rad group were significantly increased (all p<0.05; **Figures 6F-H**). These results indicate that NOTCH1 expression can reduce the radiosensitivity of cervical cancer tissues *in vivo*.

## 4 Discussion

This study focused on the regulatory role of the NOTCH signaling pathway in cervical cancer. By integrating clinical sample analysis, multi-omics technologies (single-cell RNA sequencing, high-throughput sequencing), and *in vitro* and *in vivo* experimental models, it systematically investigated the mechanism of NOTCH1 in cervical cancer progression and radiosensitivity, and clarified the targeted regulatory relationship between has-miR-449a and NOTCH1. Results showed that the activation of the NOTCH signaling pathway was significantly associated with poor prognosis in patients with cervical cancer. As a core protein of the pathway, NOTCH1 was highly expressed in tumor tissues. It could enhance the proliferative capacity of cancer cells by promoting cell cycle progression, inhibit plasma cell infiltration in the immune microenvironment, and reduce the sensitivity of cervical cancer to radiotherapy. In contrast, has-miR-449a could reverse the malignant phenotype mediated by NOTCH1 by directly targeting and inhibiting NOTCH1 expression. This study provides a new theoretical basis and potential therapeutic targets for elucidating the pathogenic mechanism of cervical cancer and optimizing radiotherapy strategies.

Aberrant activation of the NOTCH signaling pathway plays a critical role in the initiation and progression of various malignant tumors (36, 37), and this study further confirms its important value in the prognostic evaluation of cervical cancer. Analysis of 304 cervical cancer samples from the TCGA database using ssGSEA revealed that the NOTCH signaling pathway score was significantly negatively correlated with both OS and RFS of patients. To identify the core prognostic-related molecules in the pathway, univariate survival analysis and SHAP analysis were performed on NOTCH pathway genes. The results showed that NOTCH1 was positively correlated with poor prognosis in patients, and had the highest SHAP value, exerting the most significant impact on survival outcomes. Meanwhile, protein-protein interaction network analysis and correlation analysis indicated a strong association between NOTCH1 and the other 3 genes in the pathway, suggesting that NOTCH1 may be the core node regulating the activation of the NOTCH pathway. Furthermore, in the FCH cohort, patients in the NOTCH1 high-expression group had significantly shorter OS than those in the low-expression group, which further verified the clinical significance of NOTCH1 as a poor prognostic marker for cervical cancer. This is consistent with the findings of studies on NOTCH1 in colorectal cancer, ovarian cancer, breast cancer, and hematological malignancies (27, 38–40), indicating that upregulated expression of NOTCH1 may be one of the signals of poor prognosis across different types of cancers.

This study reveals the specific mechanisms by which NOTCH1 promotes cervical cancer progression from two dimensions: cell-autonomous regulation and tumor microenvironment remodeling. In terms of cell cycle regulation, *in vitro* experiments showed that cervical cancer cells with NOTCH1 knockdown exhibited significantly reduced proliferative capacity. This is consistent with the findings of studies on NOTCH1 in various tumors (41–43). NOTCH1 expression has been reported to be involved in cell cycle regulation in multiple cell types (36, 44–46), in line with this, the present study found that after NOTCH1 knockdown, the proportion of cells in the G1 phase increased, while the proportions of cells in the S phase and G2 phase decreased. These results suggest that NOTCH1 can maintain the malignant proliferative activity of cancer cells by promoting the G1-S phase transition. At the single-cell level, NOTCH1 showed the highest expression in CPA6+ malignant cells and CEL+ malignant cells, and these two cell subsets were significantly enriched in cell cycle-related pathways and classical oncogenic pathways. This indicates that NOTCH1 may drive the overall progression of cervical cancer by regulating the cell cycle process of specific malignant cell subsets.

Against the backdrop of the era of immune checkpoint blockade and adoptive T-cell therapy, the core role of T cells in anti-tumor immunity is undisputed. However, recent research evidence has shown that tumor-infiltrating B cells and plasma cells, collectively referred to as tumor-infiltrating B lymphocytes (TIL-Bs), are also key pleiotropic participants in anti-tumor responses (19). Functionally exhausted or dysfunctional CD8+ and CD4+ TILs often highly express the C-X-C motif chemokine ligand 13 (CXCL13), which is involved in B cell recruitment. This phenomenon suggests that in the face of persistent tumor existence, these T cells actively “seek” assistance from B cells (47, 48). Moreover, this interaction ultimately induces the formation of tertiary lymphoid structures (TLSs)—*de novo* lymph node-like structures emerging in the tumor stroma that actively participate in the initiation and maintenance of adaptive immune responses (49). From the perspective of the present study results, in terms of the regulation of the immune microenvironment, the proportion of plasma cells in the H_NOTCH1 group was significantly reduced, while there was no significant difference in the proportion of B cells. Pseudotime analysis further confirmed that the differentiation capacity of B cells into plasma cells was significantly impaired in the H_NOTCH1 group. Meanwhile, validation in TCGA cohort showed a significant negative correlation between the ssGSEA score of plasma cells and the expression level of NOTCH1. At the clinical prognostic level, since existing studies have confirmed that TIL-Bs have positive prognostic significance in most cancers, this directly explains the phenomenon observed in the present study—significantly shortened OS in cervical cancer patients with high NOTCH1 expression and indirectly verifies that NOTCH1 may regulate tumor prognosis by affecting TIL-B function. Furthermore, cell communication analysis at the molecular level revealed significant differences in the interaction intensity of two ligand-receptor pathways, MIF-(CD74+CXCR4) and MIF-(CD74+CD44), between malignant cells with high and low NOTCH1 expression. Based on this, it is hypothesized that NOTCH1 may hinder the differentiation and maturation of B cells into plasma cells by inhibiting MIF-related signaling pathways. This mechanistic hypothesis is consistent with the research findings of D Kitamura et al. (44), but further *in vitro* and *in vivo* experiments are still required to clarify the causal relationship.

The association between NOTCH1 and radiosensitivity has been reported in various types of cancers. Consistent with the findings of Zhang et al., the NOTCH1/HES1 signaling pathway can enhance the radiosensitivity of colorectal cancer cells (26). In another study on colorectal cancer, Roth et al. found that enhanced NOTCH signaling promotes uncontrolled cell growth and resistance to cell death in cancer, and that NOTCH1-dependent activation is mediated by inhibiting the cyclin-dependent kinase inhibitor p27 (50). In this study, NOTCH1 overexpression significantly increased the survival rate of cervical cancer cells after irradiation. This complements the mechanistic study by Roth et al., suggesting that NOTCH1-mediated radioresistance may depend on changes in the cell cycle. Has-miR-449a, as an upstream molecule of NOTCH1, exerts an enhancing effect on radiosensitivity, which is consistent with the research trends in other cancer types (51, 52). However, overexpression of has-miR-449a significantly improved the radiosensitivity of cervical cancer by inhibiting NOTCH1 expression in the current study, indicating that the mechanism of action of has-miR-449a may vary across different cancer types. Notably, IHC results showed that the expression of NOTCH1 and the Ki-67 in patients with PR after definitive radiotherapy were significantly higher than those in patients with CR. This directly links clinical efficacy to NOTCH1 expression, providing clinical evidence for the NOTCH1 axis as a predictive indicator of radiosensitivity.

While this study provides evidence for the role of NOTCH1 in cervical cancer, it still has limitations. First, in terms of cell models, this study only used HeLa and SiHa cell lines, and failed to cover rare pathological subtypes such as cervical adenosquamous carcinoma and small cell carcinoma of the cervix. Therefore, subsequent studies should incorporate multi-subtype cell lines and patient-derived organoid models to more accurately evaluate the role of NOTCH1. Second, the animal model used in this study was immunodeficient nude mice, which cannot simulate the adaptive immunity of normal human bodies. Thus, future research should establish immunocompetent models to verify the regulatory effect of NOTCH1 on humoral immunity and the potential of combining NOTCH1-targeted therapy with immunotherapy.

## Data Availability

The original contributions presented in the study are included in the article/**Supplementary Material**. Further inquiries can be directed to the corresponding authors.
