# Supplementary material for: Multi-omics reveals that NOTCH1 promotes cervical cancer progression and reduces radiosensitivity
Source: Front Immunol. 2025 Nov 11;16:1703032. doi: 10.3389/fimmu.2025.1703032 (PMC12643866; doi:10.3389/fimmu.2025.1703032)
Supplement: Supplementary file 1 [file Table1.docx]

Supplementary Material

**Supplementary tables**

Supplementary table 1. Clinical baseline table of the cervical cancer cohort.

Supplementary table 2. Primer sequences used for qPCR in this study.

Supplementary table 3. Results of Univariate and Multivariate Analysis.

**Supplementary figures**


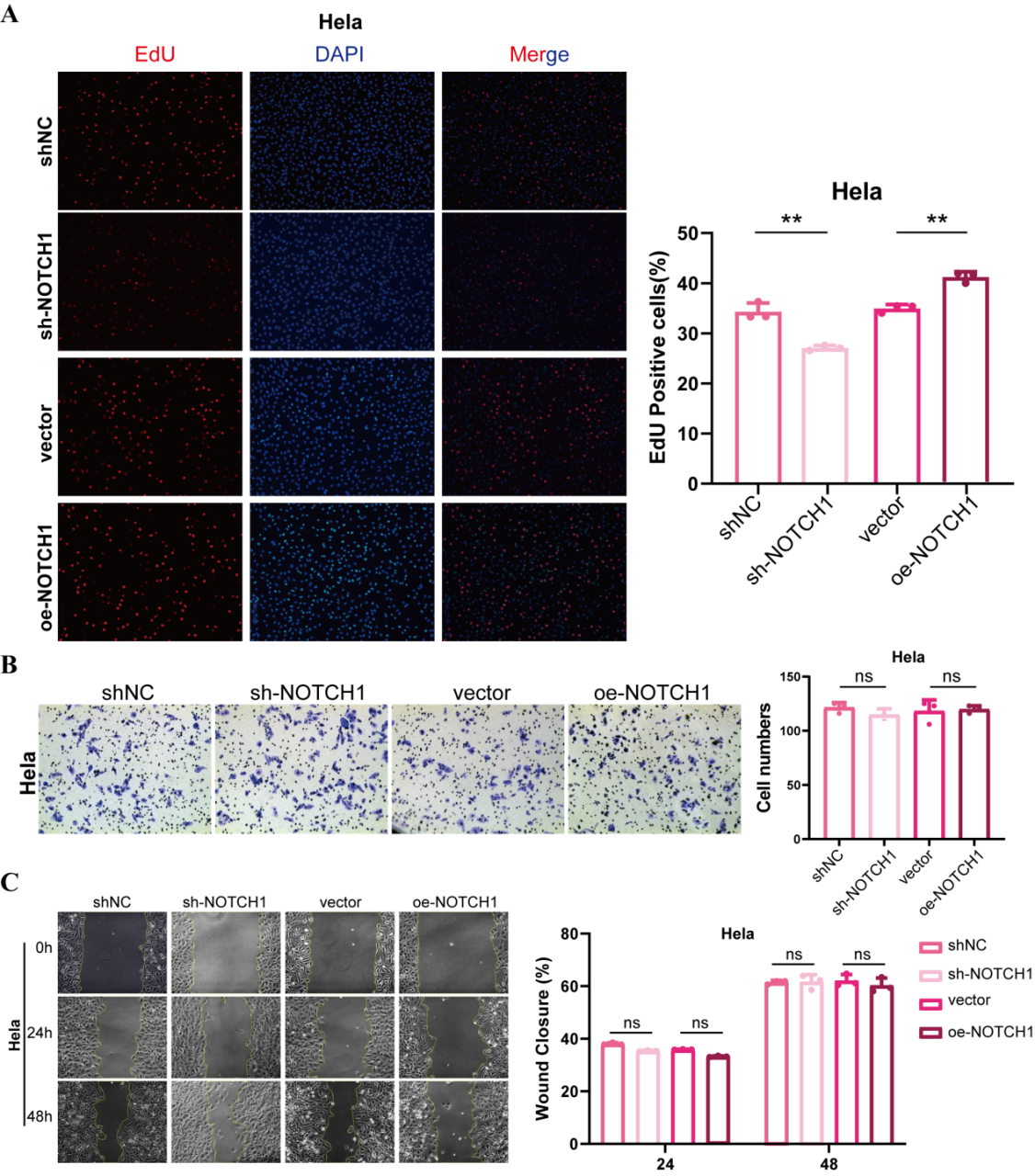


**Supplementary figure 1. The expression of NOTCH1 promotes the proliferation of Hela cells.** (A) Representative images and bar graphs of the EdU experiment show the differences in the rate of EdU positive cells among different Hela cells. (B) Representative image and bar chart show the results of the transwell assay in Hela cells. (C) Representative image and bar chart show the results of the wound healing assay in Hela cells.


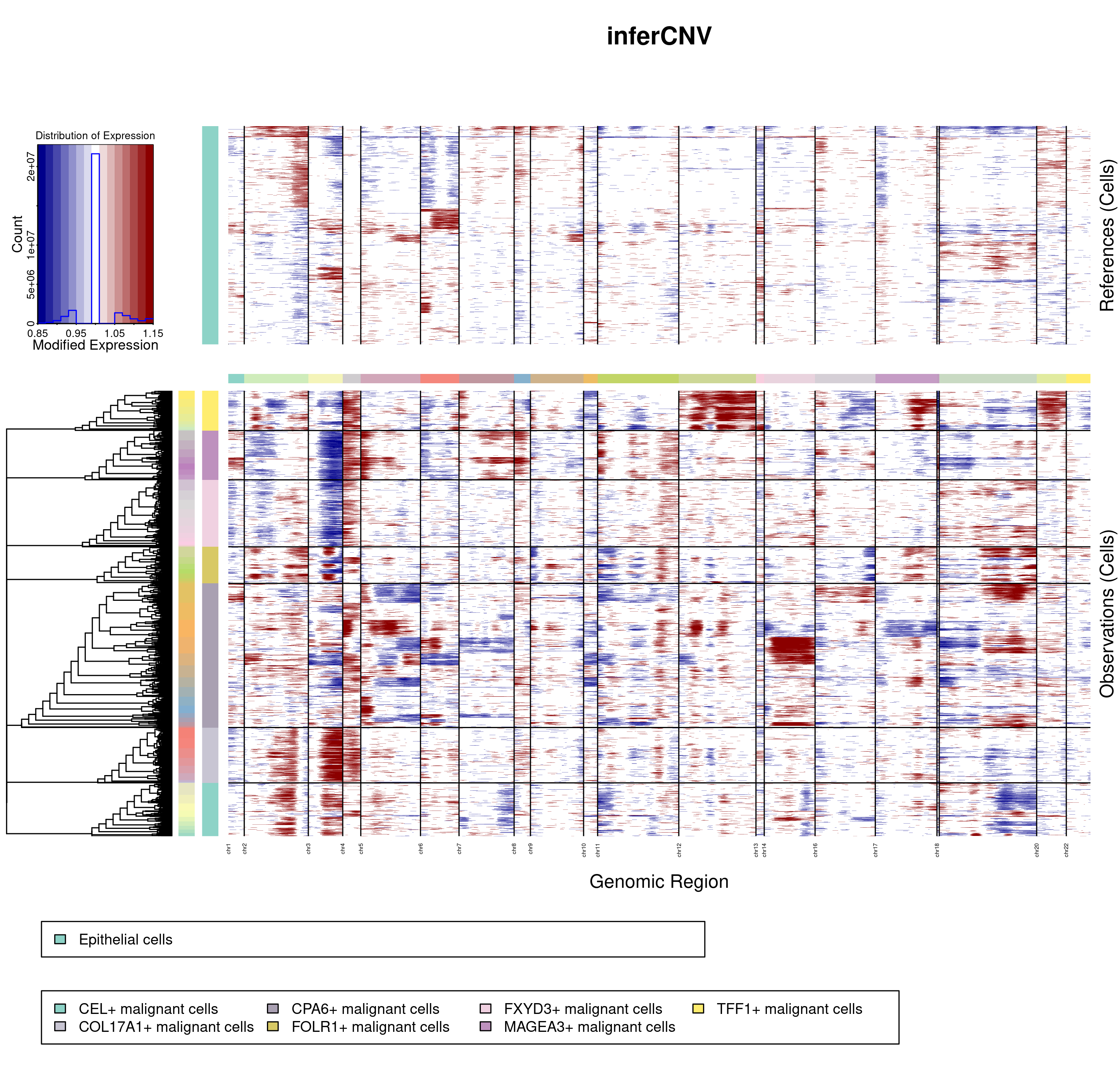
**Supplementary Figure 2. InferCNV heatmap of epithelial cells and malignant cells.**

**
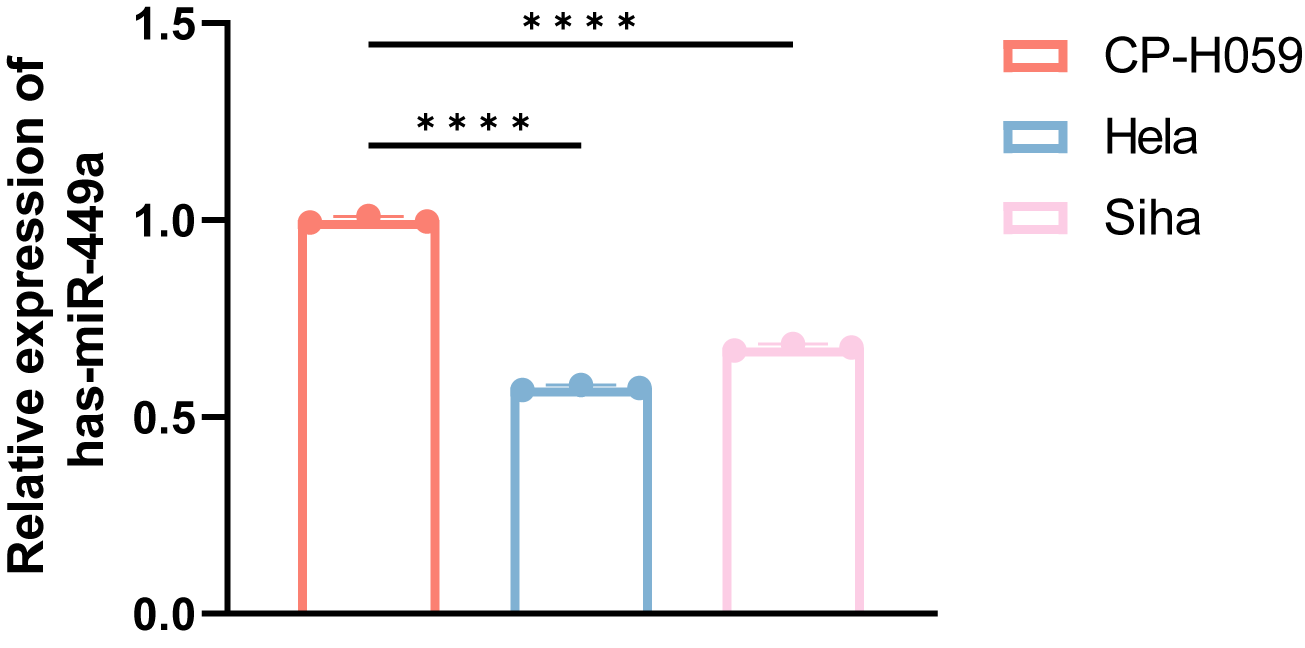
**

**Supplementary Figure 3. Bar chart showing differences in has-miR-449a expression among different cell lines.**
