# Supplementary material for: Multi-omics reveals that NOTCH1 promotes cervical cancer progression and reduces radiosensitivity
Source: Front Immunol. 2025 Nov 11;16:1703032. doi: 10.3389/fimmu.2025.1703032 (PMC12643866; doi:10.3389/fimmu.2025.1703032)
Supplement: Supplementary file 2 [file Table2.docx]

| Characteristics | Total(N) | Univariate analysis | |  | Multivariate analysis | |
| --- | --- | --- | --- | --- | --- | --- |
|  |  | Hazard ratio (95% CI) | P value |  | Hazard ratio (95% CI) | P value |
| age | 291 | 1.017 (0.999 - 1.034) | 0.063 |  | 1.015 (0.993 - 1.038) | 0.192 |
| BMI | 253 | 0.949 (0.907 - 0.993) | **0.024** |  | 0.980 (0.936 - 1.026) | 0.390 |
| histological_type | 291 |  |  |  |  |  |
| Squamous Cell Carcinoma | 241 | Reference |  |  |  |  |
| Adenocarcinoma | 45 | 0.950 (0.485 - 1.860) | 0.881 |  |  |  |
| Adenosquamous | 5 | 1.981 (0.271 - 14.485) | 0.501 |  |  |  |
| TNM_Stage | 285 |  |  |  |  |  |
| Ⅰ | 159 | Reference |  |  | Reference |  |
| Ⅱ | 64 | 0.828 (0.421 - 1.630) | 0.584 |  | 0.749 (0.334 - 1.679) | 0.482 |
| Ⅲ | 41 | 1.285 (0.637 - 2.592) | 0.483 |  | 1.216 (0.515 - 2.871) | 0.656 |
| Ⅳ | 21 | 4.512 (2.427 - 8.387) | **< 0.001** |  | 4.330 (2.048 - 9.153) | **< 0.001** |
| NRPS | 291 | 6.014 (3.074 - 11.766) | **< 0.001** |  | 4.644 (2.013 - 10.712) | **< 0.001** |
